# Case report: unprecedented case of infantile cerebral infarction following COVID-19 and favorable outcome

**DOI:** 10.3389/fimmu.2024.1357307

**Published:** 2024-03-25

**Authors:** Shuhong Zheng, Hairui Chen, Weiwei Xu, Haifeng Li, Zhongyu Chen, Jianhua Li, Enfu Tao

**Affiliations:** ^1^ Graduate School, Zhejiang Chinese Medical University, Hangzhou, Zhejiang, China; ^2^ Department of Children’s Rehabilitation, Wenling Maternal and Child Health Hospital, Wenling, Zhejiang, China; ^3^ Department of Rehabilitation, Children’s Hospital, Zhejiang University School of Medicine, National Clinical Research Center for Child Health, National Children’s Regional Medical Center, Hangzhou, Zhejiang, China; ^4^ Department of Radiology, Children’s Hospital, Zhejiang University School of Medicine, National Clinical Research Center for Child Health, National Children's Regional Medical Center, Hangzhou, Zhejiang, China; ^5^ Department of Rehabilitation Medicine, Sir Run Run Shaw Hospital, Zhejiang University School of Medicine, Hangzhou, Zhejiang, China; ^6^ Department of Neonatology and Neonatal Intensive Care Unit, Wenling Maternal and Child Health Care Hospital, Wenling, Zhejiang, China

**Keywords:** COVID-19, cerebral infarction, infant, hemiplegia, rehabilitation

## Abstract

The 2019 novel coronavirus, SARS-CoV-2, was highly prevalent in China as of December 2022, causing a range of symptoms, predominantly affecting the respiratory tract. While SARS-CoV-2 infection in children is generally mild, severe cases, especially in infants, are rare. We present a case of a previously healthy 7-month-old infant who developed cerebral infarction and coagulation dysfunction three days after COVID-19 onset. Clinically, the infant had weakness in the left limbs and pinpoint bleeding spots. A cranial magnetic resonance imaging showed ischemic strokes in the right basal ganglia and thalamus. Laboratory tests indicated thrombocytopenia and coagulation dysfunction. Inflammatory cytokines like interleukin-10 were elevated, with increased CD3^+^, CD4^+^, and CD8^+^ T lymphocytes but decreased CD3^-^ CD16^+^ CD56^+^ natural killer cells. Treatment included mannitol, dexamethasone, oral aspirin, and vitamins B1 and B6 for reducing intracranial pressure, antiinflammation, anticoagulation, and nerve support, respectively. During the recovery phase, rehabilitation therapy focused on strength training, fine motor skills, and massage therapy. The infant gradually improved and successfully recovered. While rare, such cases can lead to severe complications. These combined efforts were instrumental in achieving significant functional recovery in the patient, demonstrating that even in severe instances of pediatric cerebral infarction due to COVID-19, positive outcomes are attainable with early and comprehensive medical response.

## Introduction

COVID-19, a globally recognized issue, arises from the widely susceptible SARS-CoV-2 virus, affecting both adults and children. Clinical symptoms are varied, ranging from fever, respiratory issues, and gastrointestinal symptoms to asymptomatic or mono-symptomatic cases like loss of smell or taste. Some patients may exhibit nonspecific neurological symptoms such as headache, dizziness, and myalgia. In most pediatric cases, the process is benign (1, 2).The risk factors for ischemic stroke increase against the backdrop of COVID-19 (3, 4).While ischemic stroke is not a common complication of COVID-19, its incidence is low in adults (5, 6) and especially rare in children (7). Ischemic stroke as a complication of COVID-19 can cause varying degrees of damage in children, ranging from hemiplegia and disability to severe cases leading to death (8, 9).

Recently, there have been reports of life-threatening COVID-19 in infants, involving cases in those as young as six months and two months old (8–10). Mierzewska-Schmidt et al. reported a rare and severe case where a previously healthy two-month-old male infant developed acute hemorrhagic necrotizing encephalitis as a result of COVID-19 (10). Fraser et al. reported a clinical case where a six- month-old infant experienced rapid progression to cerebral infarctions, pneumonia, and septic shock after COVID-19. Postmortem sputum cultures indicated *Mycobacterium tuberculosis* (8). Furthermore, Ghosh et al. described a case of a 6-month-old previously healthy term boy with both miliary tuberculosis and COVID-19 co-infection developed strokes, severe sepsis, and electrolyte abnormalities, cerebral infarctions, and declined rapidly within 6 days (9). The case reports suggest that infants with COVID-19 are prone to severe illness, potentially life-threatening, especially in previously healthy infants with concurrent tuberculosis. Additionally, two of the reported cases were male infants (9) (10), and the gender was not mentioned in another case (8), indicating that male infants might also be a high-risk factor for mortality due to COVID-19. Unfortunately, despite aggressive medical intervention, these infants ultimately succumbed to their conditions.

Clinically, we presented an exceptionally rare case of a previously healthy 7-month-old infant developing ischemic stroke and cerebral infarction with hemiplegia following COVID-19. The case was promptly evaluated and managed, with successful neurological rehabilitation, leading to a good recovery.

## Case description

**Figure 1:**
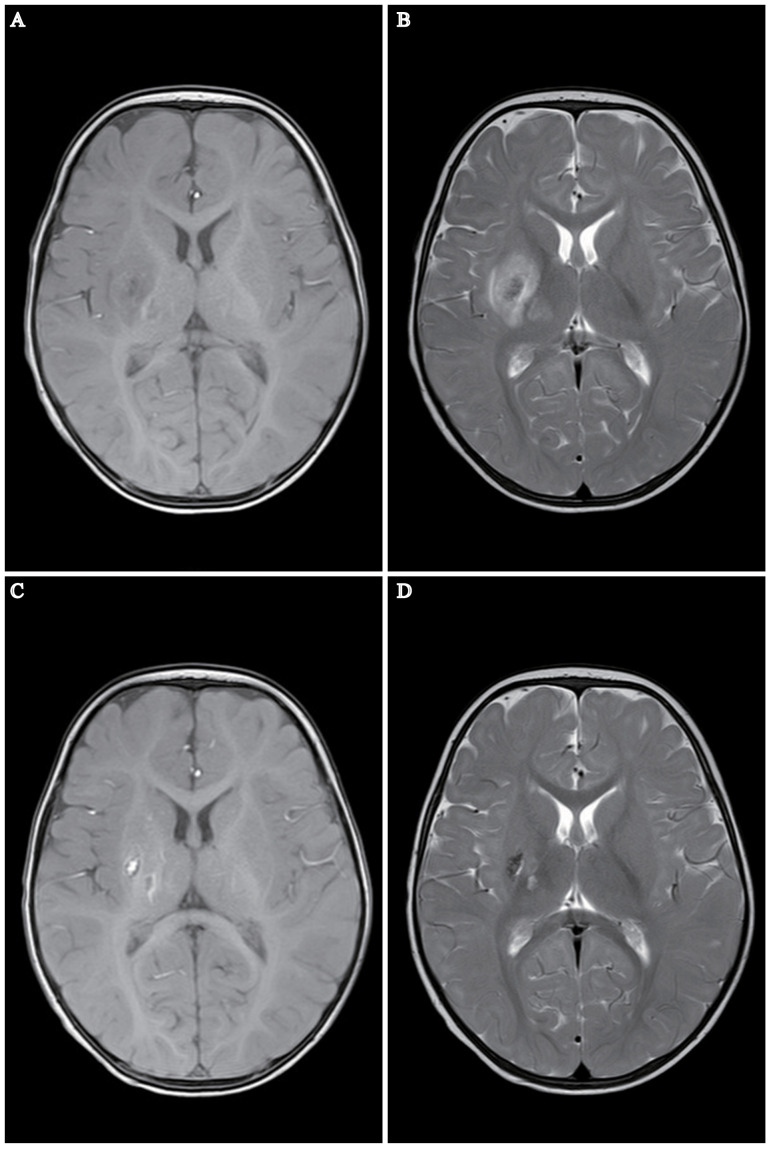
MRI Depiction of Cerebral Infarction in a 7-Month-Old Infant Post COVID-19. **(A, B)** MRI reveals a patchy region in the right basal ganglia and right thalamus exhibiting long T1 and T2 signal intensities. The internal signal is heterogeneous, predominantly displaying high signal intensity on DWI and FLAIR images, indicative of infarction in these areas. No other significant abnormal MR signal abnormalities are noted. **(C, D)** Follow-up MRI one month post-stroke. Patchy areas exhibiting long T1 and T2 signal intensities are present in the right basal ganglia and right thalamus, characterized by short T1 signals and comparatively shorter T2 signals within the lesions. No other significant abnormal MR signal anomalies are detected. Notably, regions of encephalomalacia are evident in the right basal ganglia and right thalamus, with a discernible reduction in lesion size relative to earlier assessments.

A 7-month-old male infant presented with fever following his parents’ COVID-19. A throat swab PCR test confirmed the infant as SARS-CoV-2 positive. On the third day of fever, the child suddenly exhibited left-sided limb weakness, indicating possible neurological involvement. Examination revealed neurological signs: left upper limb strength at grade I, left lower limb strength at grade III, and right limb strength at grade V. The child was alert with normal mental status, muscle tone, tendon reflexes, negative bilateral Babinski signs, and scattered pinpoint bleeding spots, and no other rashes were observed. A cranial magnetic resonance imaging (MRI) indicated right basal ganglia and thalamic infarction (**Figures 1A, B**). Neck vascular examination showed no significant abnormalities. The complete blood count (CBC) revealed a white blood cell (WBC) count of 9.69 × 10^9^/L, lymphocyte percentage of 90.2%, platelets at 89×10^9^/L, and hemoglobin at 116g/L, indicating thrombocytopenia. Coagulation tests showed fibrinogen at 1.26 g/L, plasma D-dimer at 0.16mg/L, with normal prothrombin time (PT) and activated partial thromboplastin time (APTT), suggesting coagulation dysfunction. Blood gas analysis was unremarkable. Cerebrospinal fluid (CSF) analysis revealed clear transparency, negative Pandy’s test, WBC count at 1.0 × 10^6^/L, red blood cell count at 5.0×10^6^/L. CSF culture for five days showed no bacterial or fungal growth. Biochemical analysis of CSF indicated slightly elevated glucose at 6.63 mmol/L, chloride at 127.9 mmol/L, and total protein at 345.8 mg/L, which were higher than normal. The inflammatory marker interleukin-10 (IL-10) was elevated at 6 pg/ml, while IL-2, IL-4, IL-6, tumor necrosis factor-α (TNF-α), and interferon (IFN)-γ remained within the normal range. The proportions of immune cell markers demonstrated an increase with 71.85% of CD3^+^, 40.70% of CD4^+^, and 26.55% of CD8^+^ T lymphocytes, while CD3^-^ CD16^+^ CD56^+^ natural killer cells was significantly decreased. Tests for enteroviruses, herpes simplex virus, Epstein-Barr virus, HBV virus, tuberculosis, polio, mycoplasma pneumoniae, and chlamydia yielded negative outcomes. Liver and kidney function tests, electrolytes, chest X-ray, echocardiogram, electroencephalography, and magnetic resonance angiography were all normal. Blood cultures, including those for bacteria and fungi, were negative.

The infant was diagnosed with cerebral infarction, hemiplegia, thrombocytopenia, and febrile infection. Upon admission, he received intravenous mannitol to reduce intracranial pressure, intravenous dexamethasone for anti-inflammation and swelling, oral aspirin for anticoagulation, and supportive therapy with vitamins B1 and B6, while closely monitoring vital signs. During hospitalization, the infant remained stable without seizures. Two days post-admission, his temperature normalized, and a repeat CBC showed WBC at 7.15×10^9^/L, lymphocytes at 86.6%, and platelets normalized to 191×10^9^/L. Mannitol was discontinued, and dexamethasone was stopped on the third day, continuing with aspirin and supportive therapy. The infant’s limb strength gradually improved. On the ninth day, the left upper limb strength improved to grade III, left lower limb to grade IV, and right limbs remained at grade V. Muscle tone was normal, bleeding spots receded, and coagulation function returned to normal. He was discharged for continued rehabilitation.

During rehabilitation, the infant underwent neurological tests, Bayley developmental assessment, Gross Motor Function Measure (GMFM) (**Table 1**), and Fine Motor Function Measure (FMFM) (**Table 2**). The Bayley assessment showed normal cognitive development but delayed motor skills. GMFM assessment showed delays in gross motor functions, while FMFM indicated significant differences in upper limb function between sides, with the left side markedly lagging. The infant underwent half-hour daily training in sitting, kneeling, crawling for gross motor stability and postural transition, enhancing muscle strength, stability, and coordination. Additionally, he received half-hour daily sessions of upper limb strength, grasping, coordination occupational therapy, and traditional Chinese massage to promote muscular and neural circulation and prevent muscle atrophy. After one month of rehabilitation, the infant showed marked improvement in left upper and lower limb functions, nearly reaching grade V in the left upper limb and grade V in the left lower limb, with normal muscle tone. A follow-up MRI indicated reduced size of the old lesion and resolved surrounding edema (**Figures 1C, D**). **Tables 3**, **1**, and **2** summarize the clinical course and assessment results before and after rehabilitation.

**Table 1:** Gross Motor Function Measure (GMFM-88) assessment comparison pre- and post-rehabilitation in the infant.

Items	Section A		Section B		Section C		Section D		Section E		Total percentage	
	pre	post	pre	post	pre	post	pre	post	pre	post	pre	post
Raw score	42	45	18	29	0	3	0	0	0	0	22.7	29
Functional area percentage	82	88	30	48	0	7	0	0	0	0		

A: lying and turning score, B: sitting position score, C: crawling and kneeling score, D: standing position score, E: walking and running score.

**Table 2:** Fine Motor Function Measure Assessment comparison pre- and post-rehabilitation in the infant.

Items	Pre- rehabilitation		Post- rehabilitation	
	Left	Right	Left	Right
A	15	15	15	15
B	2	15	16	16
C	0	14	13	16
D	0	8	8	12
E	0	6	6	7
Total score	17	58	58	66
Conversion of score, mean ± SD	21.54 ± 2.19	40.81 ± 1.18	40.81 ± 1.18	42.83 ± 1.14

A: visual tracking score, B: upper limb joint mobility score, C: grasping ability score, D: manipulative skills score, E: hand-eye coordination score. SD, standard deviation.

**Table 3:** Clinical course summary of the Infant with COVID-19.

Clinical course	Clinical findings	Treatment
Day 1	Fever; swab PCR test confirmed COVID-19	Symptomatic treatment
Day 3	Fever; left-sided limb weakness; neurological signs: left upper limb strength at grade I, left lower limb strength at grade III, and right limb strength at grade V, and scattered pinpoint bleeding spots; MRI: right basal ganglia and thalamic infarction; platelets at 89×10^9^/L; fibrinogen at 1.26 g/L, plasma D-dimer at 0.16mg/L; CSF: glucose at 6.63 mmol/L, chloride at 127.9 mmol/L, and total protein at 345.8 mg/L; IL-10 at 6 pg/ml; CD3^+^, CD4^+^, and CD8^+^ T lymphocytes increased; CD3^-^ CD16^+^ CD56^+^ natural killer cells decreased;	Intravenous mannitol; intravenous dexamethasone; oral aspirin; and supplement with vitamins B1 and B6; vital signs closely monitored.
Day 5	Temperature normalized; platelets normalized	
Day 6	Temperature normalized	Mannitol discontinued; dexamethasone stopped; continuing with aspirin
Day 9	The left upper limb strength improved to grade III, left lower limb to grade IV, and right limbs remained at grade V; normal muscle tone; bleeding spots receded; normal coagulation function;	Discharge
Day 10	GMFM: delays in gross motor functions, FMFM: significant differences in upper limb function between sides, with the left side markedly lagging	Rehabilitation: half-hour daily training in sitting, kneeling, crawling; half-hour daily sessions of upper limb strength, grasping, coordination occupational therapy; traditional Chinese massage
One month post- rehabilitation	Improvement in left upper and lower limb functions, nearly reaching grade V in the left upper limb and grade V in the left lower limb, with normal muscle tone; MRI: reduced size of the old lesion and resolved surrounding edema	Continuing with rehabilitation

CSF, cerebrospinal fluid; GMFM, Gross Motor Function Measure; FMFM, Fine Motor Function Measure.

## Discussion

In this case, we reported a 7-month-old infant developed an ischemic stroke resulting in cerebral infarction and coagulation dysfunction following COVID-19. To the best of our knowledge, this case represents one of the youngest patients with a favorable outcome reported for ischemic stroke secondary to COVID-19.

Cerebrovascular complications associated with COVID-19 have been documented in adults, often in those with underlying cardiovascular diseases. Acute ischemic strokes following COVID-19 are exceedingly rare in children (11–13). Most children infected with COVID-19 exhibit either asymptomatic cases, mild respiratory symptoms, or fever (14). Pneumonia is one of the most common complication following COVID-19 (15). However, in this particular case, the infant displayed symptoms of fever but notably did not exhibit any signs of pneumonia. The complications of COVID-19 in children are non-specific neurological symptoms and cerebrovascular events are very rare (7, 16). In infants, however, complications can be much more severe, sometimes resulting in life-threatening ischemic strokes (8–10). We reported a rare case of an ischemic stroke and cerebral infarction leading to hemiplegia in an infant following COVID-19.

The pathogenic mechanisms leading to cerebral infarction following COVID-19 are not yet fully elucidated. Studies indicate that COVID-19 increases the risk of ischemic stroke (17). In this case, the infant developed cerebral infarction just three days after exhibiting fever from the infection, indicating rapid disease progression. Post-infection, COVID-19 may trigger cytokine cascade reactions and endothelial cell dysfunction (18, 19), leading to an inflammatory response, thrombocytopenia, and coagulation dysfunction (20). Damage to endothelial cells and coagulation dysfunction may lead to the formation of microthrombi and the development of ischemic stroke (21). Moreover, it has been reported that cytokines and the body’s immune response are correlated with the severity or risk of COVID-19 (22, 23). In severe COVID-19 cases, patients may face acute respiratory distress syndrome, multiple organ dysfunction syndrome, or even death due to a cytokine storm. This hyperinflammatory condition is characterized by the uncontrolled release of pro-inflammatory cytokines, leading to significant complications. High levels of numerous crucial pro-inflammatory cytokines, such as interleukin-1 (IL-1), IL-2, IL-6, tumor necrosis factor-α, interferon (IFN)-γ, IL-10 and so on, have been found in severe COVID-19 (24, 25). Moreover, the hyperproduction of IL-10 and IL-6 have been linked to cytokine storm-induced mortality in fatal thrombocytopenia syndrome and severe and critically ill COVID-19 patients (26). In addition, elevated IL-6 levels in serum at the time of admission have been linked to predicting a more severe progression of COVID-19 (27). Furthermore, risk factors, such as male gender, age and pre-existing comorbidities could indicate the progression of COVID-19 into a severe and critical stage (23). Indeed, infants (six-month-old, male gender) with concurrent tuberculosis and COVID-19 may face a fatal outcome (8, 9). Recently, Mierzewska-Schmidt et al. reported a case of acute hemorrhagic necrotizing encephalitis in a previously healthy, 2-month-old male infant with SARS-CoV-2 infection (10), suggested that even in the absence of underlying diseases, SARS-CoV-2 infection in younger male infants can potentially lead to a fatal outcome. We reported a case of a 7-month-old male infant presenting with thrombocytopenia, coagulation dysfunction, and elevated cytokines, such as IL-10, indicating that our case were a severe instance of pediatric COVID-19. Notably, in our case, only IL-10 level was elevated, while other cytokines, such as IL-6, remained within normal range. This unique cytokine profile indicates a distinct immune response, offering valuable insights into the complex role of cytokines in COVID-19 and its neurological effects. Furthermore, the proportions of immune cells in our patient also underwent changes. CD3^+^, CD4^+^, and CD8^+^ T lymphocytes was significantly increased while CD3^-^ CD16^+^ CD56^+^ natural killer cells was significantly decreased, these results may be related to the severity of the disease (28). Fortunately, our case involved no underlying diseases, including the absence of tuberculosis co-infection, and our patient was relatively older compared to the three infants previously reported to have succumbed to COVID-19 (8–10). The protective factors including relative older age and no underlying comorbidities could be a significant factor in the favorable outcome of our case. Other studies indicate that hypoxemia could be a key factor in the occurrence of cerebral infarctions associated with COVID-19. Hypoxemia results in reduced oxygen supply to tissues, causing ischemia and hypoxia, and leads to the accumulation of red blood cells, increasing blood viscosity. This heightened blood viscosity can exacerbate the reduction in tissue oxygenation and nutrition, thereby perpetuating a detrimental cycle (29). A significant correlation between elevated whole blood viscosity and increased mortality rates was determined in patients with COVID-19 (30).Alterations in blood viscosity can profoundly impact blood flow dynamics, potentially tripling the risk of thrombus formation in both arteries and veins (31). Although the infant in this case exhibited respiratory symptoms due to COVID-19, there was no involvement of the lungs or signs of hypoxemia. Therefore, there is currently no basis to attribute the occurrence of post-COVID-19 cerebral infarction to hypoxemia. Furthermore, COVID-19 itself exhibits neuro-invasive and neurotropic characteristics, implying that the virus can directly or indirectly invade the central nervous system (32, 33). Several hypothesized pathways for the neuroinvasion of SARS-CoV-2 have been proposed, encompassing mechanisms like transsynaptic transfer through infected neurons, invasion through the olfactory nerves, infection of the vascular endothelium, and the migration of leukocytes across the blood-brain barrier (BBB) (34). The primary host cell receptor for the SARS-CoV-2 is the angiotensin-converting enzyme 2 (ACE2). ACE2 is expressed in both neurons and glial cells. The binding of SARS-CoV-2 to ACE2 can lead to vasoconstriction and cellular damage (35, 36). The spike protein of SARS-CoV-2 can also cause dysfunction in cerebral vascular endothelium by activating Ras homolog family member A, a key molecule involved in regulating the dynamics of endothelial cell cytoskeleton and tight junction complexes (37). This activation can subsequently compromise the integrity of BBB (38) and the blood-cerebrospinal fluid barrier (39). This might also explain why the primary manifestation in this infant post-infection was ischemic stroke. The aforementioned pathophysiological mechanisms are consistent with the clinical presentation observed in this case post-infection: an inflammatory response, a decline in platelets, scattered pinpoint bleeding spots throughout the body, coagulation dysfunction, leading to a right-sided cerebral infarction, and consequently, limb weakness and neurological changes in the left side of the body.

Additionally, this case involves right basal ganglia and thalamic infarction. Previous studies have also reported rare instances of right cerebral infarction due to COVID-19 in adolescents aged 10 and 11 years (40, 41). Furthermore, in a reported case of infant fatality due to COVID-19, similar involvement of the basal ganglia and thalamus has been documented (10). Research indicates that central nervous system infections resulting from COVID-19 predominantly present as focal lesions in medium-sized vessels, often leading to basal ganglia infarctions (42, 43). This pattern is consistent with the infarction locations observed in our case, suggesting a predilection for the basal ganglia in COVID-19-related cerebral infarctions in pediatric patients. However, some cases have reported involvement of the right middle cerebral artery and right posterior cerebral artery territories, as well as the bilateral superior cerebellar arteries (8, 44). The detailed mechanisms underlying these observations warrant further exploration. We hypothesize that the susceptibility of COVID-19 to affect these regions might be related to the abundant blood flow in these areas. The brain’s blood flow is particularly abundant in areas with high metabolic demands and dense neuronal networks. One such region is the basal ganglia, which is involved in various complex brain functions including motor control, cognition, and emotion. The basal ganglia receive a rich blood supply primarily from the branches of the middle cerebral artery, making them susceptible to ischemic events in conditions that affect cerebral circulation (45). Kaneko et al. showed that ACE2 gene expression and protein levels in human brain were progressively increased by vessel size and flow rates, indicating that brain endothelial cells are susceptible to direct SARS-CoV-2 infection through flow-dependent expression of ACE2 (46). Interestingly, Lindskog et al. revealed that ACE2 expression in the normal brain is limited to the choroid plexus and ependymal cells, with minimal expression elsewhere (47). Chen et al. leveraged public brain transcriptome databases to demonstrate ACE2 expression in specific brain regions, notably the choroid plexus and thalamus’s paraventricular nuclei. Their analysis revealed ACE2’s presence across various cell types, including neurons and non-neuronal cells such as astrocytes, oligodendrocytes, and endothelial cells, particularly in the human middle temporal gyrus and posterior cingulate cortex (48). Hernández and colleagues discovered differential expression of ACE2 in the rat brain, noting its widespread presence in brain vasculature. They observed the highest concentration of ACE2-expressing capillaries in areas critical for sensory processing and autonomic regulation, including the olfactory bulb and various hypothalamic and brainstem nuclei. Additionally, ACE2 expression was identified in astrocytes, pericytes, and endothelial cells—integral components of the blood-brain barrier (49). These studies suggest the variable expression of ACE2 in the brain could provide a pathophysiological basis for COVID-19’s invasion of neural pathways. This may also be linked to the occurrence of COVID-19-related neurological symptoms, offering insights into how the virus impacts the nervous system. Crucially, COVID-19 markedly influences ACE2 expression within the brain, notably enhancing ACE2 levels in endothelial cells, predominantly in the white matter. This upregulation is most pronounced in patients with severe neurological symptoms, suggesting a direct correlation between ACE2 levels and the intensity of COVID-19’s neurological effects (47). Therefore, it is speculated that the ACE-2 receptor may be relatively highly expressed in the right basal ganglia and thalamic regions of infants, compared to other areas of the brain. This may help elucidate why reports of infant cerebral infarction cases due to COVID-19, including our own, commonly involve the basal ganglia and thalamus. Certainly, further research is necessary to validate this assertion. Interestingly, Heine et al. have recently found that specific structural imaging alterations in the thalamus and basal ganglia are associated with the prolonged fatigue experienced by patients suffering from post-COVID syndrome (50). This serves as further significant evidence of COVID-19 affecting the basal ganglia and hypothalamus.

Specific predictors for early detection of cerebral infarction as a complication of COVID-19 may enhance the prognosis of affected children. It has been reported that a significant increase in Factor VIII is associated with the hypercoagulable state related to COVID-19 (51). Additionally, significantly elevated levels of D-dimer and decreased fibrinogen can serve as early warning indicators (52). In our case, despite normal D-dimer levels, cerebral infarction still occurred, suggesting that normal D-dimer levels alone cannot completely rule out the risk of stroke. This might require a combined assessment with fibrinogen levels, where normal D-dimer levels could be misleading. Routine tests including CBC, platelet count, PT, APTT, and fibrinogen are helpful in identifying potential risk in patients (53). Therefore, for infants with persistent fever exceeding three days and any abnormalities in platelet counts, heightened vigilance for hypercoagulability and the potential for cerebral infarction is warranted. Timely coagulation function tests, and when necessary, early utilization of cerebral and neurovascular imaging to assess the possibility of stroke, can have a positive impact on the prognosis of the disease.

In treatment, anticoagulants, anti-inflammatory therapies, antiviral drugs, angiotensin-converting enzyme inhibitors, and angiotensin receptor blockers are recommended. Early use of aspirin is associated with lower mortality rates (54). In this case, our treatment encompassed acute phase management with measures to reduce intracranial pressure, anti-inflammatory and anticoagulation therapies, alongside supportive treatments (55). During recovery, we employed rehabilitation training (56, 57) combined with traditional Chinese massage therapy (58, 59). Furthermore, it should be highlighted that, as per the latest consensus statement from Chinese experts on diagnosing, treating, and preventing COVID-19 in children, there are currently no specific antiviral drugs authorized for pediatric patients with SARS-CoV-2 infection in China (60). Consequently, despite the case’s severity, antiviral medications were not utilized. One month into rehabilitation, significant improvements were observed in the infant’s left upper and lower limb functions, with left upper limb strength nearly reaching grade V and left lower limb strength at grade V, while right limb strength remained at grade V, and muscle tone was normal. Assessments using the GMFM and FMFM showed marked improvements post-rehabilitation, and follow-up cranial MRI indicated significant resolution of the lesions. This suggests that prompt treatment in the acute phase, coupled with effective rehabilitation during recovery, can markedly enhance the prognosis of infantile patients suffering from COVID-19-induced basal ganglia and thalamic infarction.

Notably, this report has several limitations. Firstly, it is based on a single case study. Secondly, there was a lack of focused attention on infants presenting with persistent fever exceeding three days post COVID-19. This oversight led to missed opportunities in monitoring coagulation function changes and delayed the identification of cerebral infarction risks. Prompt intervention at this stage might have mitigated the risk of subsequent cerebral infarction, despite fever being a prevalent symptom in COVID-19 cases.

## Conclusion

This case emphasizes the need for vigilant monitoring and swift intervention in young COVID-19 patients, especially infants with prolonged fever, due to the risk of serious complications like cerebral infarction. Successful management involved prompt treatments for reducing intracranial pressure and inflammation, as well as ongoing rehabilitation, highlighting their key roles in recovery. The case demonstrates that even in severe instances of pediatric cerebral infarction following COVID-19, early and comprehensive medical care can lead to positive outcomes.

## Data availability statement

The original contributions presented in the study are included in the article/supplementary material. Further inquiries can be directed to the corresponding authors.

## Ethics statement

The studies involving humans were approved by Medical Ethics Committee of Wenling Maternal and Child Health Care Hospital. The studies were conducted in accordance with the local legislation and institutional requirements. Written informed consent for participation in this study was provided by the participants’ legal guardians/next of kin. The manuscript presents research on animals that do not require ethical approval for their study. Written informed consent was obtained from the individual(s) for the publication of any potentially identifiable images or data included in this article.

## Author contributions

SZ: Data curation, Formal analysis, Methodology, Resources, Funding acquisition, Writing – original draft, Writing – review & editing. HC: Data curation, Investigation, Validation, Writing – review & editing. WX: Data curation, Investigation, Validation, Writing – review & editing. HL: Data curation, Investigation, Validation, Writing – review & editing. ZC: Data curation, Investigation, Validation, Writing – review & editing. JL: Conceptualization, Methodology, Supervision, Writing – review & editing, Investigation. ET: Conceptualization, Methodology, Supervision, Writing – review & editing, Formal analysis, Validation, Visualization, Writing – original draft.
